# Correlations between α-Linolenic Acid-Improved Multitissue Homeostasis and Gut Microbiota in Mice Fed a High-Fat Diet

**DOI:** 10.1128/mSystems.00391-20

**Published:** 2020-11-03

**Authors:** Xiaoyu Gao, Songlin Chang, Shuangfeng Liu, Lei Peng, Jing Xie, Wenming Dong, Yang Tian, Jun Sheng

**Affiliations:** a Engineering Research Center of Development and Utilization of Food and Drug Homologous Resources, Ministry of Education, Yunnan Agricultural University, Kunming, Yunnan, People’s Republic of China; b College of Food Science and Technology, Yunnan Agricultural University, Kunming, Yunnan, People’s Republic of China; c Yunnan Provincial Key Laboratory of Biological Big Data, Yunnan Agricultural University, Kunming, Yunnan, People’s Republic of China; d Yunnan Provincial Engineering Research Center for Edible and Medicinal Homologous Functional Food, Yunnan Agricultural University, Kunming, Yunnan, People’s Republic of China; e Key Laboratory of Pu-er Tea Science, Ministry of Education, Yunnan Agricultural University, Kunming, Yunnan, People’s Republic of China; Vanderbilt University Medical Center

**Keywords:** α-linolenic acid, microbiota, homeostasis, polyunsaturated fatty acid, obesity

## Abstract

Previous studies have shown that α-linolenic acid (ALA) has a significant regulatory effect on related disorders induced by high-fat diets (HFDs), but little is known regarding the correlation between the gut microbiota and disease-related multitissue homeostasis. We systematically investigated the effects of ALA on the body composition, glucose homeostasis, hyperlipidemia, metabolic endotoxemia and systemic inflammation, white adipose tissue (WAT) homeostasis, liver homeostasis, intestinal homeostasis, and gut microbiota of mice fed an HFD (HFD mice). We found that ALA improved HFD-induced multitissue metabolic disorders and gut microbiota disorders to various degrees. Importantly, we established a complex but clear network between the gut microbiota and host parameters. Several specific differential bacteria were significantly associated with improved host parameters. *Rikenellaceae*_RC9_gut_group and *Parasutterella* were positively correlated with HFD-induced “harmful indicators” and negatively correlated with “beneficial indicators.” Intriguingly, *Bilophila* showed a strong negative correlation with HFD-induced multitissue metabolic disorders and a significant positive correlation with most beneficial indicators, which is different from its previous characterization as a “potentially harmful genus.” *Turicibacter* might be the key beneficial bacterium for ALA-improved metabolic endotoxemia, while *Blautia* might play an important role in ALA-improved gut barrier integrity and anti-inflammatory effects. The results suggested that the gut microbiota, especially some specific bacteria, played an important role in the process of ALA-improved multitissue homeostasis in HFD mice, and different bacteria might have different divisions of regulation.

**IMPORTANCE** Insufficient intake of *n*-3 polyunsaturated fatty acids is an important issue in modern Western-style diets. A large amount of evidence now suggests that a balanced intestinal microecology is considered an important part of health. Our results show that α-linolenic acid administration significantly improved the host metabolic phenotype and gut microbiota of mice fed a high-fat diet, and there was a correlation between the improved gut microbiota and metabolic phenotype. Some specific bacteria may play a unique regulatory role. Here, we have established correlation networks between gut microbiota and multitissue homeostasis, which may provide a new basis for further elucidating the relationship between the gut microbiota and host metabolism.

## Abstract (precis)

Insufficient intake of *n*-3 polyunsaturated fatty acids is an important issue in modern Western-style diets. A large amount of evidence now suggests that a balanced intestinal microecology is considered an important part of health. Our results show that α-linolenic acid administration significantly improved the host metabolic phenotype and gut microbiota of mice fed a high-fat diet, and there was a correlation between the improved gut microbiota and metabolic phenotype. Some specific bacteria may play a unique regulatory role. Here, we have established correlation networks between gut microbiota and multitissue homeostasis, which may provide a new basis for further elucidating the relationship between the gut microbiota and host metabolism.

## INTRODUCTION

In the past few decades, whether in the Eastern countries represented by China or the Western countries represented by the United States, dietary habits have changed dramatically. Modern diets that focus on fast, stimulating taste are rich in saturated fatty acids and *n*-6 polyunsaturated fatty acids (PUFAs), while *n*-3 PUFAs are relatively insufficient (1–3). This change in diet is a risk factor for many noncommunicable diseases such as obesity, diabetes, metabolic syndrome, nonalcoholic fatty liver disease, cardiovascular disease, osteoporosis, cognitive impairment, and even cancer (4). Increased consumption of *n*-6 PUFAs, which are abundant in Western diets, contributes to obesity and related diseases. A high-fat diet (HFD) can cause systemic, multitissue metabolic disorders, which in turn cause a range of health problems. Hence, HFD animal models are increasingly being used to simulate the potential risks of Western diets.

α-Linolenic acid (ALA), an essential fatty acid needed for human health, is commonly found in some vegetable oil seeds. Perilla frutescens, Salvia hispanica, and flax (Linum usitatissimum) are rich in ALA. ALA is the precursor of longer-chain *n*-3 fatty acids such as eicosapentaenoic acid (EPA) and docosahexaenoic acid (DHA) and has a variety of nutraceutical and pharmacological effects, including cardiovascular-protective, neuroprotective, anti-cardiovascular-disease, anticancer, antineuropathy, antiosteoporotic, anti-inflammatory, and antioxidative effects (5).

A balanced intestinal microecology is now considered an important part of health. The gut microbiota is associated with a number of health problems, especially metabolic syndrome, diabetes, and obesity, which are closely related to dietary factors. In recent years, a series of studies reported the effects of *n*-3 PUFAs or related carriers rich in *n*-3 PUFAs on the gut microbiota of animals or humans, including healthy volunteers (6), overweight individuals with metabolic syndrome (7), breast cancer survivors (8), mice with alcohol-induced liver injury (9), obese mice (4), early-life antibiotic exposure-induced obese mice (10), rats fed a high-fat diet (11), individuals with nonalcoholic fatty liver disease (12), and early-life-stress rats (13). Most of these studies focus on the effects of EPA and DHA on the gut microbiota of animals and humans with related diseases, and there are few reports on the effect of ALA monomers on the gut microbiota (14–16).

Although existing research reports have shown that ALA and ALA-rich diets have significant regulatory effects on related diseases induced by high-fat diets, gut microbes that play a key role in improving high-fat-diet-related diseases need to be identified, and correlation networks between the gut microbiota and multitissue homeostasis need to be established. To this end, the effects of ALA on the body composition, glucose homeostasis, hyperlipidemia, metabolic endotoxemia and systemic inflammation, white adipose tissue (WAT) homeostasis, liver homeostasis, intestinal homeostasis, and gut microbiota of mice fed a high-fat diet were studied systematically in this study.

## RESULTS

### Effects of ALA administration on body composition and energy intake in mice.

**FIG 1:**
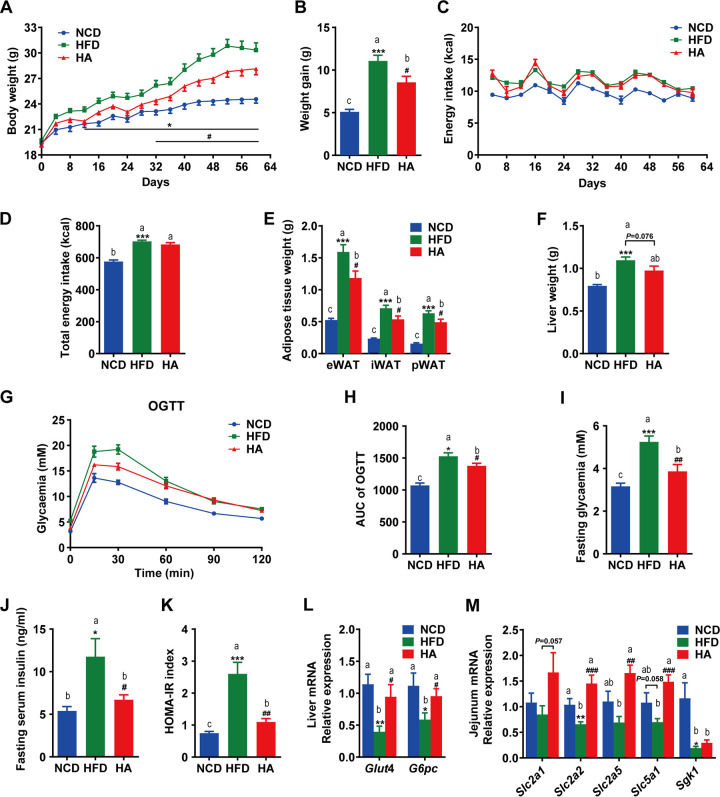
Effects of ALA administration on body composition, energy intake, and glucose metabolism. (A and B) Body weight changes. (C and D) Energy intake. (E) Epididymal (eWAT), inguinal (iWAT), and perirenal (pWAT) weights. (F) Liver weight. (G) Oral glucose tolerance test (OGTT). (H) Area under the concentration-time curve (AUC) for the OGTT. (I to K) Fasting glucose, fasting insulin, and homeostasis model assessment of insulin resistance (HOMA-IR) index. (L) Relative mRNA expression levels of glucose transporter 4 (*Glut4*) and glucose-6-phosphatase (*G6pc*) in the liver. (M) Relative mRNA expression levels of the glucose transporter genes glucose transporter protein type 1 (*Slc2a1*), glucose transporter protein type 2 (*Slc2a2*), glucose transporter protein type 5 (*Slc2a5*), sodium-glucose cotransporter 1 (*Slc5a1*), and glucocorticoid-regulated kinase 1 (*Sgk1*) in the jejunum. The data are expressed as means ± SEM (*n *= 10 to 12 [A to K] and *n *= 6 [L and M]). *, compared with the NCD group; #, compared with the HFD group (using the unpaired two-tailed Student *t* test). *, *P ≤ *0.05; **, *P ≤ *0.01; ***, *P ≤ *0.001; #, *P ≤ *0.05; ##, *P ≤ *0.01; ###, *P ≤ *0.001. Data with different superscript letters are significantly different (*P ≤ *0.05) according to *post hoc* one-way ANOVA.

Daily oral administration of ALA (500 mg/kg of body weight [BW]) prevented diet-induced weight gain from day 32 onward (Fig. 1A and B), and these findings were not related to changes in energy intake (Fig. 1C and D). ALA prevented fat accretion in white adipose tissue, including epididymal, perirenal, and inguinal fat depots (eWAT, pWAT, and iWAT, respectively) (Fig. 1E). Additionally, liver weight was reduced in mice of the HFD-ALA (HA)-treated group but not significantly (*P = *0.076) (Fig. 1F). These results indicate that ALA could prevent HFD-induced weight gain and obesity, which are not related to changes in energy intake.

### ALA administration improves HFD-induced glucose metabolism disorders.

After 9 weeks of treatment, we found that ALA enhanced glucose clearance (Fig. 1G and H) by the oral glucose tolerance test (OGTT). These results indicate that ALA administration improved HFD-induced glucose intolerance. Meanwhile, ALA significantly reduced the fasting glucose (FG) (Fig. 1I) and fasting insulin (INS) (Fig. 1J) levels, resulting in improved insulin resistance (IR), as suggested by the lower homeostasis model assessment of insulin resistance (HOMA-IR) index (Fig. 1K). Interestingly, ALA significantly enhanced the mRNA expression levels of glucose transporter 4 (*Glut4*) and glucose-6-phosphatase (*G6pc*) in the liver (Fig. 1L) and significantly enhanced the mRNA levels of the glucose transporter genes sodium-glucose cotransporter 1 (*Slc5a1*), glucose transporter protein type 2 (*Slc2a2*), and glucose transporter protein type 5 (*Slc2a5*) (Fig. 1M). These results suggest that ALA might improve glucose tolerance and IR by promoting glucose transport and gluconeogenesis in mice fed an HFD (HFD mice).

### ALA administration improves WAT homeostasis and hyperlipidemia in HFD mice.

**FIG 2:**
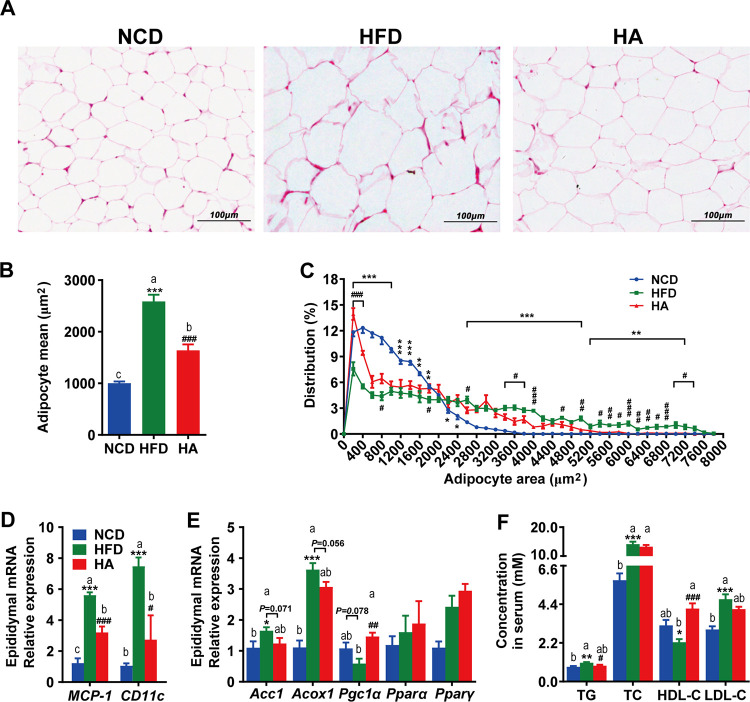
ALA improves HFD-induced lipid metabolism disorders in eWAT and serum. (A to E) Effects of ALA administration on adipocyte morphology and relative mRNA expression levels of lipogenesis, lipid oxidation, and inflammatory factors in eWAT. (A) H&E staining of paraffin sections of eWAT. (B) Adipocyte mean area (square micrometers). (C) Cell size profiling of adipocytes from eWAT. (D and E) mRNA expression levels of markers of lipogenesis (*Acc1*) and lipid oxidation (*Acox1*, *Pgc1*α, *Ppar*α, and *Ppar*γ) (E) and inflammatory factors, including *MCP-1* and *CD11c* (D). (F) Serum lipid profile. The data are expressed as means ± SEM (*n *= 8 [B to E] and *n *= 10 to 12 [F]). *, compared with the NCD group; #, compared with the HFD group (using the unpaired two-tailed Student *t* test). *, *P ≤ *0.05; **, *P ≤ *0.01; ***, *P ≤ *0.001; #, *P ≤ *0.05; ##, *P ≤ *0.01; ###, *P ≤ *0.001. Data with different superscript letters are significantly different (*P ≤ *0.05) according to *post hoc* one-way ANOVA.

To investigate whether the reduced WAT amount (Fig. 1E) was due to differences in adipocyte volumes, we measured the size distribution of eWAT cells. Compared to the group fed a normal chow diet (NCD), mice in the HFD group had increased numbers of large adipocytes and decreased numbers of small adipocytes in their eWAT, while ALA treatment reversed these changes (Fig. 2A to C). Subsequently, we investigated whether ALA could affect lipogenesis and lipid oxidation in eWAT. Compared to the NCD group, the mRNA expression levels of markers of lipogenesis (*Acc1*) and lipid oxidation (*Acox1* and *Pgc1*α) in eWAT were altered by the HFD, while ALA treatment reversed these mRNA expression levels to different extents (Fig. 2E). Meanwhile, ALA administration also reduced the mRNA expression of inflammatory factors in the eWAT of HFD mice (Fig. 2D).

Moreover, ALA improved HFD-induced hyperlipidemia. As depicted in Fig. 2F, ALA significantly reduced serum triglyceride (TG) levels and enhanced high-density lipoprotein cholesterol (HDL-C) levels (*P < *0.05). The total cholesterol (TC) and low-density lipoprotein cholesterol (LDL-C) levels were also reduced but not significantly (*P > *0.05).

### ALA administration attenuates fatty liver, metabolic endotoxemia, and systemic inflammation.

**FIG 3:**
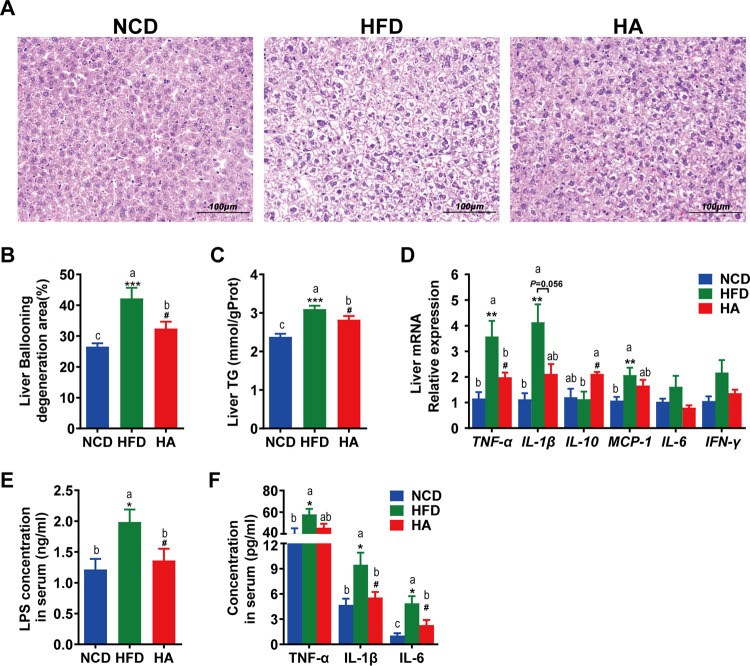
ALA attenuates HFD-induced fatty liver, metabolic endotoxemia, and systemic and hepatic inflammation. (A) Photomicrographs of H&E-stained liver sections. (B) Ballooning degeneration area (percent) in the liver. (C) TG content in the liver. (D) Relative mRNA expression levels of the liver inflammatory factors monocyte chemotactic protein 1 (*MCP-1*), *TNF-*α, *IL-6*, *IL-1*β, *IFN*-γ, and *IL-10*. (E) Serum LPS. (F) Serum TNF-α, IL-6, and IL-1β. The data are expressed as means ± SEM (*n *= 8 [A to D] and *n *= 10 to 12 [E and F]). *, compared with the NCD group; #, compared with the HFD group (using the unpaired two-tailed Student *t* test). *, *P ≤ *0.05; **, *P ≤ *0.01; ***, *P ≤ *0.001; #, *P ≤ *0.05. Data with different superscript letters are significantly different (*P ≤ *0.05) according to *post hoc* one-way ANOVA.

As shown in Fig. 3A to C, ALA attenuated the extent of HFD-induced ballooning degeneration and reduced hepatic TG accumulation. Meanwhile, ALA regulated the mRNA expression of liver inflammatory factors, suggesting that ALA improved HFD-induced hepatic inflammation, as indicated by the lower mRNA levels of tumor necrosis factor alpha (*TNF*-α) and the strong tendency toward reduced interleukin-1β (IL-1β) (*P = *0.052) (Fig. 3D). It is worth mentioning that the anti-inflammatory factor *IL-10* was significantly upregulated by ALA.

The high serum levels of lipopolysaccharide (LPS), TNF-α, IL-6, and IL-1β in the HFD group indicate that the HFD triggered metabolic endotoxemia and systemic inflammation. Importantly, ALA administration significantly reduced the serum levels of LPS, IL-6, and IL-1β (Fig. 3E and F).

### ALA remodels intestinal homeostasis in HFD mice.

**FIG 4:**
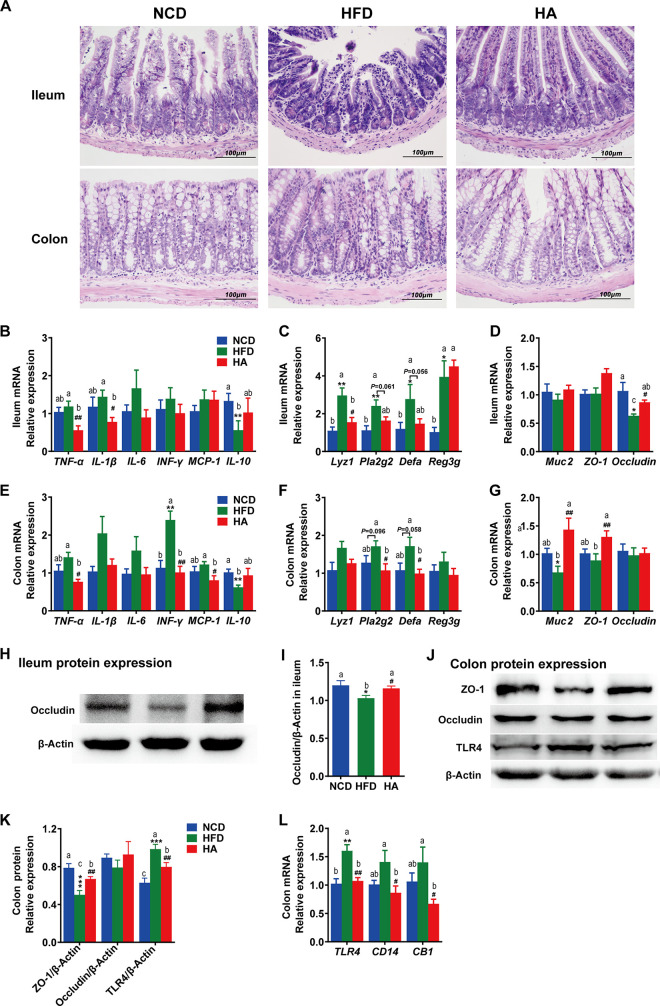
Effects of ALA administration on intestinal homeostasis in mice. (A) Photomicrographs of H&E-stained distal ileum and proximal colon sections. Infiltration of inflammatory cells into the tissue is shown in blue in the photomicrographs in the HFD group. (B and E) Expression of inflammation genes in the ileum (B) and the colon (E), including *IFN*-γ, *MCP-1*, *TNF*-α, *IL-1*β, *IL-6*, and *IL-10*. (C and F) Expression of AMP genes in the ileum (C) and colon (F), including *Defa*, *Lyz1*, *Reg3g*, and *Pla2g2*. (D and G) Expression of intestinal barrier genes in the ileum (D) and colon (G), including *Muc2*, *Occludin*, and *ZO-1*. (H to K) Expression of the intestinal barrier protein occludin in the ileum (H and I) and expression of occludin, ZO-1, and TLR4 in the colon (J to K). (L) Relative mRNA expression levels of *TLR4*, *CD14*, and *CB1* in the colon. The data are expressed as means ± SEM (*n *= 8 [B to L]). *, compared with the NCD group; #, compared with the HFD group (using the unpaired two-tailed Student *t* test). *, *P ≤ *0.05; **, *P ≤ *0.01; ***, *P ≤ *0.001; #, *P ≤ *0.05; ##, *P ≤ *0.01. Data with different superscript letters are significantly different (*P ≤ *0.05) according to *post hoc* one-way ANOVA.

The intestinal mucosa, local and systemic immune factors, and gut microbial content are important interaction factors for maintaining intestinal homeostasis. ALA treatment reduced HFD-induced intestinal inflammatory responses (Fig. 4A), including the infiltration of inflammatory cells and the mRNA expression of proinflammatory factors (Fig. 4B and E). Although the mRNA expression of proinflammatory factors in the ileum was not significantly enhanced by the HFD, *TNF-*α and *IL-1*β were significantly reduced by ALA to levels lower than those in the NCD group. The situation in the colon was different. The mRNA expression of interferon gamma (*IFN-*γ) in the colon was significantly enhanced by the HFD, and ALA significantly reduced *TNF-*α and monocyte chemotactic protein 1 (*MCP-1*) to levels lower than those in the NCD group. Notably, the anti-inflammatory factor *IL-10* in both the ileum and colon was significantly reduced by the HFD, and ALA significantly reversed it in the colon.

Antimicrobial peptides (AMPs) in the intestine are important to defend against pathogens and maintain microbiota-host homeostasis. The mRNA expression levels of AMPs, including α-defensins (*Defa*), lysozyme C (*Lyz1*), regenerating islet-derived 3-gamma (*Reg3g*), and phospholipase A2 group II (*Pla2g2*), in the ileum (Fig. 4C) were significantly upregulated by HFD challenge (*P < *0.05), but only *Lyz1* was significantly downregulated by ALA treatment in HFD mice (*P < *0.05). *Defa* and *Pla2g2* were also reduced but not significantly (*P > *0.05). In the colon (Fig. 4F), *Defa*, *Lyz1*, *Pla2g2*, and *Reg3g* were all upregulated by HFD challenge but not significantly (*P > *0.05). However, *Defa* and *Pla2g2* were significantly reduced by ALA treatment in HFD mice (*P < *0.05).

*Muc2* and *Occludin* in the colon were significantly downregulated by HFD challenge (*P < *0.05), while both were significantly upregulated by ALA treatment (Fig. 4G) (*P* > 0.05), suggesting that ALA could protect against HFD-induced impaired intestinal barrier function and intestinal homeostasis. The Western blot results for the Occludin and zona occludens protein 1 (ZO-1) proteins also proved this (Fig. 4H to K).

Toll-like receptor 4 (TLR4), cluster of differentiation 14 (CD14), and cannabinoid receptor 1 (CB1) also play important roles in the maintenance of homeostasis in the intestinal commensal system and are closely related to intestinal inflammation and barrier function. Bacterial LPS can bind to TLR4, and CD14 may be required during the binding process. We found that the HFD challenge induced high mRNA expression levels of *TLR4*, *CD14*, and *CB1*, and ALA significantly inhibited these HFD-induced high expression levels (Fig. 4L). It is worth mentioning that both the gene and protein expression levels of TLR4 were significantly upregulated by HFD challenge, while ALA significantly inhibited such high expression levels (Fig. 4L). This is consistent with the result that we measured for LPS in the circulatory system (Fig. 3E). Taken together, we believe that ALA can restore intestinal homeostasis to a certain extent by improving HFD-induced intestinal inflammation, inhibiting antibacterial peptide overexpression, and repairing the intestinal barrier.

### Effect of ALA on gut microbiota in HFD mice.

**FIG 5:**
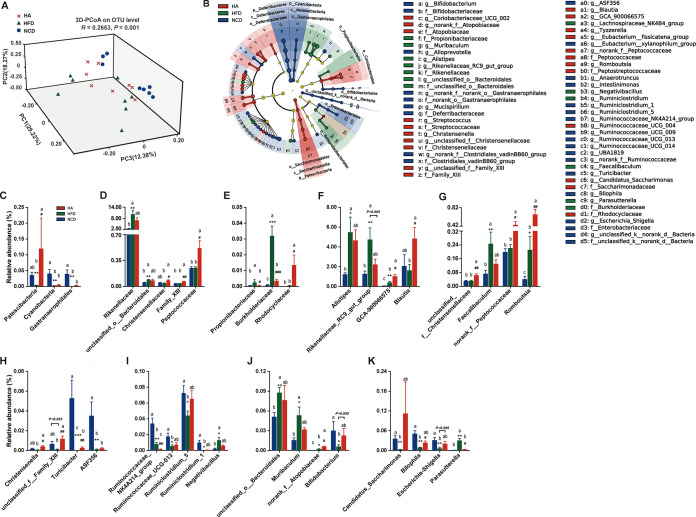
ALA restores the HFD-induced gut microbial community structural and compositional shift. (A) 3D-PCoA (principal-coordinate analysis) plot based on Bray-Curtis distance. (B) Linear discriminant analysis effect size (LEfSe) analyses (LDA score of ≥2.0). (C to K) Relative abundances of gut microbiota at the phylum, family, and genus levels, respectively, which were significantly affected by the HFD or ALA, especially those reversed by ALA treatment. The data are expressed as means ± SEM (*n *= 8 [C to K]). *, compared with the NCD group; #, compared with the HFD group (using the unpaired two-tailed Student *t* test). *, *P ≤ *0.05; **; *P ≤ *0.01; ***, *P ≤ *0.001; #, *P ≤ *0.05; ##, *P ≤ *0.01; ###, *P ≤ *0.001. Data with different superscript letters are significantly different (*P ≤ *0.05) according to *post hoc* one-way ANOVA.

To evaluate the effect of ALA on the gut microbiota of HFD mice, we sequenced the V3-V4 regions of the 16S rRNA gene. Through systematic bioinformatics analysis, we found that ALA restored the HFD-induced gut microbial community structural and composition shifts. Although the effect of ALA on alpha diversity was limited (see Fig. S1A to C in the supplemental material), ALA altered the beta diversity of HFD mice (Fig. 5A). Moreover, ALA changed the cecum microbial composition of HFD mice (Fig. S1D to F), and the microbial composition of ALA-treated mice was clustered with that of the NCD group (Fig. S1G to I).

10.1128/mSystems.00391-20.2 FIG S1 Effect of ALA on the gut microbiota of mice. (A) Sobs rarefaction curve. (B and C) Alpha diversity at the OTU level. (B) Sobs index. (C) Shannon index. (D to F) Cecum microbial composition. (D) Phylum level. (E) Family level. (F) Genus level. (G to I) Cluster heat maps of the gut microbiota in different groups. *, compared with the NCD group using the unpaired two-tailed Student *t* test. ***, *P ≤ *0.001. Download FIG S1, DOCX file, 0.6 MB. Copyright © 2020 Gao et al. 2020 Gao et al. This content is distributed under the terms of the Creative Commons Attribution 4.0 International license.

To define which bacterium might be the main bacterium responsible for the impact of ALA on HFD mice, linear discriminant analysis (LDA) effect size (LEfSe) analyses were used to obtain the dominant microbiota at different levels for each group (Fig. 5B and Fig. S2A to D). Here, a total of 79 different taxa from the 3 groups are displayed, including 4 phyla, 4 classes, 9 orders, 18 families, and 44 genera (Fig. S2A to C). Therefore, many different taxa also reflect the strong influence of the HFD and ALA on the cecum microbiota. We focused on the taxa that were significantly affected by HFD or ALA, especially those reversed by ALA treatment. At the phylum level (Fig. 5C), the relative abundances of *Patescibacteria* and *Cyanobacteria* were significantly reduced by HFD challenge, and ALA reversed these changes. Notably, the relative abundance of *Patescibacteria* was significantly increased by ALA. At the family level, the HFD significantly increased the abundances of *Rikenellaceae*, *unclassified_o_Bacteroidales*, *Propionibacteriaceae*, and *Burkholderiaceae* (Fig. 5D and E), while ALA reduced their abundances to various degrees. *Propionibacteriaceae* and *Burkholderiaceae* (Fig. 5E) were significantly decreased to levels close to those of the NCD group (*P < *0.05). Interestingly, although the HFD showed limited effects on the relative abundances of *Christensenellaceae*, *Family_XIII*, *Peptococcaceae*, and *Rhodocyclaceae*, ALA treatment significantly enhanced their abundances (*P < *0.05).

10.1128/mSystems.00391-20.3 FIG S2 Linear discriminant analysis effect size (LEfSe) analyses (LDA score of ≥2.0). (A to C) LEfSe analyses based on the NCD, HFD, and HA groups. (A) At the phylum, class, and order levels. (B) At the family level. (C) At the genus level. (D) LEfSe analyses based on the HFD and HA groups, from the phylum level to the genus level. Download FIG S2, DOCX file, 0.4 MB. Copyright © 2020 Gao et al. 2020 Gao et al. This content is distributed under the terms of the Creative Commons Attribution 4.0 International license.

At the genus level, the relative abundances of 25 genera belonging to different phyla are shown in Table S1. Compared with the NCD group, the HFD significantly increased the relative abundances of *Alistipes*, *Rikenellaceae_RC9_gut_group*, *GCA-900066575*, *Faecalibaculum*, and *Romboutsia* (*P < *0.05) (Fig. 5F and G). However, they were influenced by ALA differently. ALA inhibited the HFD-induced high relative abundances of *Alistipes*, *Rikenellaceae_RC9_gut_group*, and *Faecalibaculum* but significantly increased the relative abundances of *GCA-900066575* and *Romboutsia* (*P < *0.05). Although *Blautia*, *unclassified_f_Christensenellaceae*, and *norank_f*_*Peptococcaceae* were less affected by HFD challenge, ALA significantly increased their relative abundances in HFD mice (*P < *0.05) (Fig. 5F and G).

10.1128/mSystems.00391-20.5 TABLE S1 Primer sequences used for quantitative PCR analysis of gene expression. Download Table S1, DOCX file, 0.02 MB. Copyright © 2020 Gao et al. 2020 Gao et al. This content is distributed under the terms of the Creative Commons Attribution 4.0 International license.

Compared with the NCD group, the HFD also reduced the relative abundances of many genera of *Firmicutes* (Fig. 5H and I), including *Christensenella*, *unclassified_f_Family_XIII*, *Turicibacter*, *ASF356*, *Ruminococcaceae_NK4A214_group*, *Ruminococcaceae_UCG-013*, *Ruminiclostridium_5*, and *Ruminiclostridium_1*. Among these genera, ALA significantly reversed the low relative abundances of *Christensenella*, *unclassified_f_Family_XIII*, and *Turicibacter* in HFD mice (Fig. 5H) while further reducing the abundance of *Ruminococcaceae_NK4A214_group* (Fig. 5I).

Similar to *Alistipes* and *Rikenellaceae_RC9_gut_group* (belonging to *Bacteroidetes*), the abundances of *unclassified_o_Bacteroidales* and *Muribaculum* (genera of *Bacteroidetes*) were also significantly increased by HFD challenge (*P < *0.05) and decreased by ALA treatment (Fig. 5J). *norank_f_Atopobiaceae* and *Bifidobacterium* are the two genera of *Actinobacteria*. Both the HFD and ALA significantly promoted the abundance of *norank_f_Atopobiaceae* in mice (*P < *0.05) (Fig. 5J). Importantly, the HFD significantly inhibited the famous probiotic *Bifidobacterium* (*P < *0.05), while ALA markedly restored its abundance to a level close to that of the NCD group (*P > *0.05). Moreover, *Candidatus_Saccharimonas* (belonging to *Patescibacteria*), *Bilophila*, and *Escherichia-Shigella* (genera of *Proteobacteria*) were all significantly inhibited by the HFD, but all were markedly promoted by ALA treatment (Fig. 5K). Notably, the effects of HFD and ALA treatments on *Parasutterella* (genus of *Proteobacteria*) were exactly the opposite of those on *Bilophila*.

### Correlations between specific gut bacteria and core host parameters.

**FIG 6:**
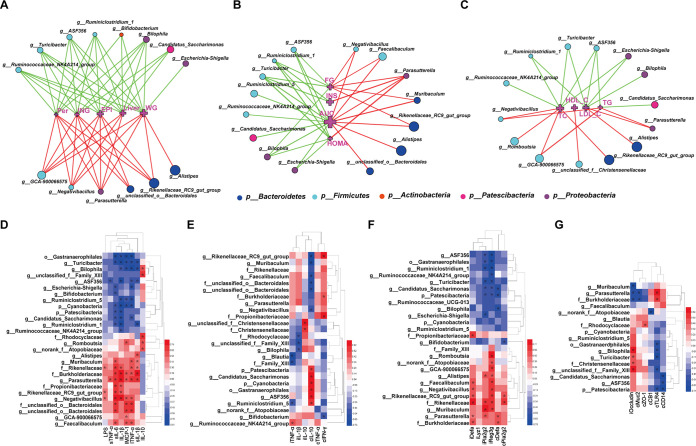
Network and heat maps showing correlations between specific gut bacteria and core host parameters. (A to C) Two-factor correlation network analysis (*P ≤ *0.05; Spearman’s *r* coefficient |*r*| of ≥0.5 [*n *= 8 in each group]). Red lines represent *r* values greater than or equal to 0.5, and green lines represent *r* values less than or equal to −0.5. (D to G) Bivariate correlations (Spearman’s *r* coefficient *r* ≥ 0.5 or *r* ≤ −0.5 [*n* = 8 in each group]). (A) Correlation between gut bacteria and body composition. WG, body weight gain; Liver, liver weight; ING, weight of inguinal fat; PER, weight of perinephric fat; EPI, weight of epididymal fat. (B) Correlation between gut bacteria and glucose hemostasis. FG, fasting glucose; INS, insulin; AUC, area under the OGTT curve; HOMA, HOMA-IR index. (C) Correlation between gut bacteria and blood lipid profile of TC, TG, HDL-C, and LDL-C in serum. (D) Correlation between gut bacteria and metabolic endotoxemia and systemic and hepatic inflammation. LPS, sTNF-α, sIL-6, and sIL-1β indicate LPS, TNF-α, IL-6, and IL-1β in serum, respectively; lIL-1β, lIL-10, lMCP-1, and lTNF-α indicate the mRNA expression levels of *IL-1*β, *IL-10*, *MCP-1*, and *TNF*-α in liver, respectively. (E) Correlation between gut bacteria and intestinal inflammation. iTNF-α, iIL-1β, and iIL-10 indicate the mRNA expression levels of *TNF*-α, *IL-1*β, and *IL-10* in the ileum, respectively; cIL-10, cTNF-α, and cIFN-γ indicate the mRNA expression levels of *IL-10*, *TNF*-α, and *IFN*-γ in the colon, respectively. (F) Correlation between gut bacteria and intestinal AMPs. iDefa, iLyz1, iPla2g2, and iReg3g indicate the mRNA expression levels of *Defa*, *Lyz1*, *Pla2g2*, and *Reg3g* in the ileum, respectively; cDefa and cPla2g2 indicate the mRNA expression levels of *Defa* and *Pla2g2* in the colon, respectively. (G) Correlation between gut bacteria and gut barrier-related indicators. iOccludin indicates the mRNA expression of *Occludin* in the ileum; cMuc2, cZO-1, cCB1, cTLR4, and cCD14 indicate the mRNA expression levels of *Muc2*, *ZO-1*, *CB1*, *TLR4*, and *CD14* in the colon, respectively. The color at each intersection indicates the value of the *r* coefficient; *P* values were adjusted for multiple testing according to the Bonferroni and Hochberg procedures. * indicates a significant correlation between these two parameters (*P ≤ *0.05).

Based on the significant improvement in multitissue homeostasis and gut microbiota of HFD mice by ALA, two-factor correlation network analysis (Fig. 6A to C) and bivariate correlation analysis (Fig. 6D to G) were used to establish the correlations between specific gut bacteria and core host parameters. As shown in Fig. 6A, correlations between specific gut bacteria (genus level) and body composition are tight and clearly visible. *Alistipes*, *Rikenellaceae_RC9_gut_group*, and *unclassified_o_Bacteroidales* (belonging to *Bacteroidetes*) showed significant and positive correlations with body composition. Genera of *Firmicutes* showed some differentiation. *GCA-900066575* and *Negativibacillus* were positively correlated with body composition, while the other four genera of *Firmicutes* (*Ruminococcaceae_NK4A214_group*, *ASF356*, *Turicibacter*, and *Ruminiclostridium_1*) were the opposite. Both *Parasutterella* and *Bilophila* are genera of *Proteobacteria*, and *Parasutterella* was positively correlated with all five parameters of body composition. However, *Bilophila* was completely the opposite. Notably, *Bifidobacterium* (belonging to *Actinobacteria*) and “*Candidatus* Saccharimonas” (belonging to *Patescibacteria*) showed negative correlations with body composition (Fig. 6A and Fig. S3A).

10.1128/mSystems.00391-20.4 FIG S3 Heat maps showing the correlations between specific gut bacteria and core host parameters. (A to C) Bivariate correlations (Spearman’s *r* coefficient *r* ≥ 0.5 or *r* ≤ −0.5; *n *= 8 in each group). (A) Correlation between gut bacteria and body composition. WG, body weight gain; Liver, liver weight; ING, weight of inguinal fat; PER, weight of perinephric fat; EPI, weight of epididymal fat. (B) Correlation between gut bacteria and glucose hemostasis. FG, fasting glucose; INS, insulin; AUC, area under the OGTT curve; HOMA, HOMA-IR index. The relative mRNA expression levels of the glucose transporter genes glucose transporter protein type 2 (*Slc2a2*), glucose transporter protein type 5 (*Slc2a5*), and sodium-glucose cotransporter 1 (*Slc5a1*) are shown. (C) Correlation between gut bacteria and lipid metabolism markers TG, TC, HDL-C, and LDL-C in serum, the mRNA expression levels of markers of lipid oxidation (*Acox1* and *Pgc1*α), and inflammatory factors (*MCP-1* and *CD11c*). The color at each intersection indicates the value of the *r* coefficient; *P* values were adjusted for multiple testing according to the Bonferroni and Hochberg procedures. * indicates a significant correlation between these two parameters (*P ≤ *0.05). Download FIG S3, DOCX file, 0.8 MB. Copyright © 2020 Gao et al. 2020 Gao et al. This content is distributed under the terms of the Creative Commons Attribution 4.0 International license.

Correlations between specific gut bacteria and blood glucose homeostasis are shown in Fig. 6B (genus level) and Fig. S3B. *Rikenellaceae_RC9_gut_group* showed a significant and positive correlation with FG and the area under the concentration-time curve (AUC) and a negative correlation with liver *Glut4* and jejunum *Slc2a2*. *Parasutterella* showed a significant and positive correlation with FG, AUC, INS, and HOMA and a negative correlation with liver *Glut4*. *Bilophila* showed exactly the opposite correlations. In addition, *Bilophila* was the only genus that was positively correlated with liver *G6pc*. Many genera of *Firmicutes* showed significant and positive correlations with FG, AUC, INS, and HOMA, especially *Turicibacter* and *Ruminiclostridium_1*; however, *Faecalibaculum* and *Negativibacillus* were positively correlated with FG and AUC.

Specific gut bacteria of different taxa were also commonly associated with lipid metabolism in both blood and eWAT (Fig. 6C and Fig. S3C). The noticeable groups are *Cyanobacteria*, *Gastranaerophilales*, *Turicibacter*, *ASF356*, *unclassified_f_Christensenellaceae*, and *Parasutterella. Cyanobacteria*, *Gastranaerophilales*, *Turicibacter*, and *ASF356* showed strong negative correlations with TC, TG, and LDL-C in serum and *Acox1*, *MCP-1*, and *CD11c* in eWAT. *Parasutterella* was basically the opposite. Notably, *unclassified_f_Christensenellaceae* was the only genus that was significantly and positively related to HDL-C.

Correlations between specific gut bacteria and metabolic endotoxemia and systemic and hepatic inflammation are shown in Fig. 6D. *Rikenellaceae*, *Burkholderiaceae*, *Rikenellaceae_RC9_gut_group*, *Negativibacillus*, and *Parasutterella* were positively correlated with LPS, TNF-α, IL-6, and IL-1β in serum and *IL-1*β, *MCP-1*, and *TNF*-α in liver. *Patescibacteria*, *Cyanobacteria*, *Gastranaerophilales*, *Turicibacter*, *g_ASF356*, *Bilophila*, and *Candidatus_Saccharimonas* showed almost the opposite correlations. Of particular interest is the correlation of these specific gut bacteria with LPS. *Rikenellaceae*, *Burkholderiaceae*, *Rikenellaceae_RC9_gut_group*, and *Negativibacillus* were significantly positively related to LPS. *Turicibacter* was the only genus that was significantly negatively related to LPS.

Correlations between specific gut bacteria and intestinal hemostasis are shown in Fig. 6E to G. A number of specific gut bacteria were significantly associated with the mRNA expression of intestinal inflammatory factors (Fig. 6E). Among them, *IL-10* in the colon was the most noticeable parameter. *Rikenellaceae*, *Rikenellaceae_RC9_gut_group*, *unclassified_o_Bacteroidales*, *Muribaculum*, and *Faecalibaculum* were significantly negatively related to *IL-10* in the colon. *Patescibacteria*, *Cyanobacteria*, *Gastranaerophilales*, *Candidatus_*Saccharimonas, *ASF356*, and *Ruminiclostridium_5* showed exactly the opposite correlations. Many specific gut bacteria have also shown a significant correlation with the gene expression of intestinal AMPs (Fig. 6F). *Reg3g* and *Pla2g2* were more related to bacteria than other AMPs. *Alistipes*, *Faecalibaculum*, *Negativibacillus*, *Rikenellaceae*, *Rikenellaceae_RC9_gut_group*, and *Parasutterella* were significantly and positively related to *Reg3g* and *Pla2g2*, while *Patescibacteria*, *Gastranaerophilales*, *ASF356*, *Ruminiclostridium_1*, *Turicibacter*, *Candidatus_Saccharimonas*, and *Bilophila* showed the opposite correlations. Moreover, *Alistipes*, *Faecalibaculum*, *Negativibacillus*, *Rikenellaceae*, *Rikenellaceae_RC9_gut_group*, and *Parasutterella* were also positively related to *Defa* in the ileum. Correlations between specific gut bacteria and gut barrier-related indicators are shown in Fig. 6G. *Parasutterella* and *Burkholderiaceae* were negatively correlated with *Occludin* in the ileum and *Muc2* in the colon but positively correlated with *TLR4* in the colon. *Christensenellaceae* and *unclassified_f_Family_XIII* showed the opposite correlations. *Patescibacteria*, *Gastranaerophilales*, *Bilophila*, *Turicibacter*, and *ASF356* were positively related to *Occludin* in the ileum but negatively correlated with *TLR4* in the colon. Notably, *Blautia* was positively related to *Muc2* in the colon, and the *Rhodocyclaceae* family was positively related to *ZO-1* in the colon. Taken together, these results constitute a complex but clear network between gut microbiota and host parameters and explain the systemic effects of ALA on HFD mice to some extent.

## DISCUSSION

Excessive growth of WAT, abnormal glucose metabolism, and lipid metabolism disorders are the basic physiological phenomena induced by a high-fat diet. Previous studies have shown that ALA or ALA-enriched diets alter body composition, improve glucose tolerance, and attenuate IR (17–19). Supplementation of ALA improves serum adiponectin levels and insulin sensitivity in patients with type 2 diabetes (20). The results of these previous studies are basically consistent with our findings. Moreover, we also found through correlation analysis that the improvement of the body composition of HFD mice by ALA may be attributed to the combined effects of several specific gut bacteria. Therefore, those specific gut bacteria that were significantly altered by ALA and significantly correlated with body composition parameters deserve more attention, especially those that were significantly reversed. *Rikenellaceae_RC9_gut_group*, *Parasutterella*, *Turicibacter*, *Bilophila*, and *Bifidobacterium* are their typical representatives at the genus level (Fig. 5F, H, J, and K).

Glucose homeostasis plays an important role in the normal function of various physiological functions of the animal body. Increasing evidence has linked impaired glycemic control and insulin resistance to the specific gut microbiota composition. Specific gut bacteria that were significantly altered by ALA and significantly correlated with parameters of glucose metabolism were also found in this study. *Rikenellaceae_RC9_gut_group*, *Parasutterella*, *Turicibacter*, and *Bilophila* are the four genera most closely related to glucose metabolism parameters.

Previous studies have reported that ALA-rich carriers show significant lipid-lowering activity, including reducing liver fat accumulation, alleviating liver steatosis, and lowering blood lipid levels (21–23). This is basically consistent with our findings. In addition, our results showed that ALA can significantly reduce the average area size of WAT cells in HFD mice. *Turicibacter* and *Parasutterella* were the two important genera that were significantly altered by ALA and significantly correlated with parameters of lipid metabolism in this study. It is suggested that *Turicibacter* and *Parasutterella* may regulate lipid metabolism in mice fed a high-fat diet. It has been shown that high-fat feeding modulates the gut microbiota, which strongly increases intestinal permeability, leading to lipopolysaccharide (LPS) absorption and metabolic endotoxemia that triggers inflammation and metabolic disorders (24, 25). We also detected HFD-induced metabolic endotoxemia in mice; inflammation of the circulatory system, liver, and adipose and intestinal tissues; and impaired intestinal barrier function. ALA improved hyperlipidemia and WAT homeostasis in HFD mice (Fig. 2), attenuated HFD-induced metabolic endotoxemia and systemic and multitissue inflammation (Fig. 3), altered the gut microbial community structure and composition of HFD mice, and remodeled intestinal homeostasis (Fig. 4 and 5).

Few studies have reported the effects of ALA on metabolic endotoxemia. ALA-enriched linseed oil supplementation may aid in the prophylaxis of endotoxemia in horses (26). Therefore, the inhibitory effect of ALA on LPS production in HFD mice found in our study is innovative and significant because the trigger factor for ALA to improve inflammation and intestinal barrier-related indicators in HFD mice may be the reduced LPS (outer membrane component of Gram-negative bacteria). Our correlation analysis results show that *Rikenellaceae_RC9_gut_group*, *Burkholderiaceae* (the family of the genus *Parasutterella*), *Turicibacter*, and *Bilophila* were significantly altered by ALA and correlated with parameters of metabolic endotoxemia and systemic and hepatic inflammation. It is worth mentioning again that *Turicibacter* was the only genus that was significantly and negatively related to LPS.

At present, some studies have reported the effects of *n*-3 PUFAs on intestinal homeostasis-related factors, including intestinal inflammation, intestinal antimicrobial peptides, and the intestinal barrier (27, 28). However, few studies have reported the effects of ALA on intestinal homeostasis-related factors. According to Zeng and coworkers, optimal dietary alpha-linolenic acid/linoleic acid ratios could improve gill immunity and strengthen the physical barrier of juvenile fish (Ctenopharyngodon idella), while the triggers of these improved immune indicators and physical barriers and their correlation with the gut microbiota are still unknown (29). We not only found that the ALA monomer remodeled intestinal homeostasis (Fig. 4) and altered the structure and composition of the gut microbial community (Fig. 5) of HFD mice but also established correlations between the indicators of intestinal homeostasis and the gut microbiota (Fig. 6).

Consistent with the above-mentioned correlations, similar microbial groups showed strong correlations with core indicators of intestinal inflammation, intestinal AMPs, and intestinal barriers. Specifically, *Rikenellaceae_RC9_gut_group* was significantly and negatively related to *IL-10* in the colon; *Rikenellaceae_RC9_gut_group*, *Burkholderiaceae* (the family of the genus *Parasutterella*), *Turicibacter*, and *Bilophila* were significantly related to *Reg3g* and *Pla2g2*; *Parasutterella* was negatively correlated with *Occludin* in the ileum and *Muc2* in the colon but positively correlated with *TLR4* in the colon; and *Bilophila* and *Turicibacter* were positively related to *Occludin* in the ileum but negatively correlated with *TLR4* in the colon. Notably, *Blautia* was positively related to *Muc2* in the colon.

Overall, we believe that *Rikenellaceae_RC9_gut_group*, *Parasutterella*, *Turicibacter*, and *Bilophila* may play more important roles in the process of ALA-improved multitissue homeostasis in HFD mice, while other taxa might play a synergistic role with these four genera in various ways. This potential synergy may be systemic evidence that ALA improves multitissue homeostasis in HFD mice by reducing metabolic endotoxemia. Many previous reports can help us to prove that these taxa have such functions, and many other studies have also reported the potential correlation between these taxa and core host parameters.

*Rikenellaceae_RC9_gut_group* is a dominant group of *Bacteroidetes*. A few special reports have focused on this group. However, existing research suggests that this group may have some specific functions. The significantly increased abundance of *Rikenellaceae_RC9_gut_group* may be associated with the decreased serum levels of TG and TC in the early life of female offspring by maternal dietary genistein (30). The significant alteration of *Rikenellaceae_RC9_gut_group* may contribute to the pathogenesis of acute myocardial ischemia by impacting intestinal permeability, oxidative stress, and energy metabolism (31). The abundance of *Rikenellaceae_RC9_gut_group* impacted the interaction between vitamin A and TLR4 (32). In our study, this group was generally positively correlated with HFD-induced “harmful indicators” and negatively correlated with “beneficial indicators.” These findings collectively reflect the vital role of this potentially harmful bacterium in HFD-induced health problems.

The genus *Parasutterella* (*Proteobacteria*) has been defined as a core component of the healthy human and mouse gut microbiota and has been correlated with various health outcomes (33), including inflammatory bowel disease (IBD) (34), obesity (35, 36), diabetes (37), fatty liver disease (38), chronic kidney disease (39), major depression (40), Henoch-Schönlein purpura in children (41), cholestasis in infants (42), and Hashimoto’s thyroiditis in patients (43). A reduction of *Parasutterella* in response to an HFD has been observed in both animal models and human studies, indicating a positive correlation between *Parasutterella* abundance and HFD-induced metabolic phenotypes (44–46). Our results also showed that the abundance of *Parasutterella* increased significantly after the induction of the HFD, but ALA reversed this change. Notably, a previous study of flaxseed oil (rich in ALA) improving alcoholic fatty liver also found similar phenomena. Through correlation analysis, we found that *Parasutterella* is the most striking genus, which is positively correlated with almost all harmful indicators in HFD mice and negatively correlated with all beneficial indicators. ALA may play a system-improving role in HFD mice by inhibiting *Parasutterella*.

*Bilophila* is widely regarded as a potentially harmful genus or a conditional pathogen and is also considered an LPS-producing and intestinal sulfate-reducing bacterium (47). Bilophila wadsworthia aggravates high-fat-diet-induced metabolic dysfunctions in mice (48). A human stool-derived Bilophila wadsworthia strain caused systemic inflammation in specific-pathogen-free mice (49). Increased *Bilophila* abundances have been associated with liver damage (50). However, the results of previous studies are also contradictory. Some studies have reported that a decrease in *Bilophila* was related to the occurrence of the disease. For example, a decreased abundance of the genus *Bilophila* was evident at the inflamed sites of patients with ulcerative colitis (UC) compared with the corresponding sites of non-IBD controls (51). In addition, stachyose showed a selective enrichment of *Bilophila* in improving HFD/streptozotocin-induced inflammation in rats with type 2 diabetes (51). Our results also show that ALA can significantly restore the abrupt decline in *Bilophila* caused by an HFD. *Bilophila* showed a strong negative correlation with HFD-induced multitissue metabolic disorders and a significant positive correlation with most of the beneficial indicators. Therefore, we believe that the function of *Bilophila* might be conditional and complex, and it is worthy of further analysis and research.

*Turicibacter* is a genus of the phylum *Firmicutes* with a variety of biological activities. Studies have shown that *Turicibacter* is related to a series of diseases, including diet-induced obesity (52, 53), autism spectrum disorder (54), lymphoma (55), difficulty in defecation (56), hypertension (57), and Parkinson’s disease (58). The abundance of *Turicibacter* was closely related to lipid metabolism in rats fed a high-fat diet (11, 59) and type 2 diabetic rats (60). Random blood glucose in obese rats was significantly and negatively correlated with the abundance of *Turicibacter* (61). *Turicibacter* also has immunomodulatory (62) and inflammation-suppressive (63) effects. The results of these previous studies are consistent with our findings. In addition, *Turicibacter* was the only genus that was significantly and negatively related to LPS in this study. We speculate that *Turicibacter* may be the key beneficial bacterium for ALA-improved metabolic endotoxemia in HFD mice.

*Blautia* is a dominant genus of the *Firmicutes*. Although the HFD did not significantly reduce its abundance, ALA treatment significantly increased its abundance in HFD mice. In the analysis of the results of LEfSe, *Blautia* was an important factor for distinguishing the HFD and HA groups. Our correlation analysis results also show that *Blautia* was strongly positively correlated with the mRNA expression of the colonic mucus marker protein Muc2 and negatively correlated with colon and colonic *IFN*-γ and ileal *TNF*-α. This suggests that *Blautia* might play an important role in the ALA-mediated improvement in gut barrier integrity and anti-inflammatory effects. Previous research also indicated that *Blautia* might have diverse biological activities. *Blautia* was associated with reduced mortality from graft-versus-host disease (64), metabolic results (obesity and reduced liver steatosis) observed in humanized obese mice (65), and visceral fat accumulation in adults (66). When a variety of exogenous substances were found to improve steatohepatitis and the gastrointestinal barrier (67) and nonalcoholic fatty liver (68) and reduce obesity and maintain intestinal barrier integrity (69) in mice, the abundance of *Blautia* increased significantly. These rich previous studies corroborate our findings.

In summary, we systematically studied the influence of ALA monomers on HFD-induced obesity-related host parameters and the gut microbiota. We found that ALA could significantly improve HFD-induced multitissue homeostasis. Meanwhile, the established correlation networks between the gut microbiota and multitissue homeostasis in HFD mice lay a foundation for further clarifying the relationship between the gut microbiota and host metabolism, which will be a good model of the “gut-derived doctrine of chronic diseases.”

## MATERIALS AND METHODS

### Materials.

α-Linolenic acid (ALA) (97%) was obtained from Shanghai Guchen Biological (Shanghai, China). A normal chow diet (NCD) (catalog number AIN-93M) and a 60% high-fat diet (catalog number TP23400) were purchased from Trophic Animal Feed High-tech Co., Ltd., Nantong, China. Triglycerides (TG), serum total cholesterol (TC), low-density lipoprotein cholesterol (LDL-C), high-density lipoprotein cholesterol (HDL-C), and liver TG were measured using kits from the Nanjing Jiancheng Bioengineering Institute (Nanjing, China). Plasma insulin was assessed using an ultrasensitive enzyme-linked immunosorbent assay (ELISA) kit (Alpco, USA). Serum lipopolysaccharide (LPS) levels were quantified using an ELISA kit (Cusabio, USA). Serum TNF-α, IL-6, and IL-1β were measured using enzymatic kits purchased from Beijing 4A Biotech Co., Ltd. (Beijing, China). Antibodies were purchased from Abcam (occludin, catalog number ab167161), Thermo Fisher (ZO-1, catalog number 61-7300), Wanleibio (TLR4, catalog number WL00196), and Sino Biological (β-actin, catalog number SB100166-MM10).

### Animals and treatment.

C57BL/6J male mice aged 5 weeks (*n* = 36) (15 to 18 g; Chengdu Dossy Experimental Animals Co., Ltd., China) were bred in the animal facility of Yunnan Agricultural University. The animals were housed in a controlled environment (24°C ± 1°C in a 12-h daylight cycle with lights off at 20:00 h) with *ad libitum* access to food and water. After 1 week of acclimatization on an NCD, the mice were fasted overnight (12 h) for the determination of fasting blood glucose levels. Subsequently, the mice were divided into three groups of 12 mice each according to their body weights and fasting glucose levels (70). Treatment started concomitantly with the introduction of an HFD and consisted of daily oral doses (500 mg/kg·BW) of ALA. The mice in the control groups received the vehicle (water) for 9 weeks. Body weight gain and food intake were assessed once every 4 days. At week 9, animals were sacrificed in chambers saturated with CO_2_. The animal protocol used in this study was reviewed and approved by the Institutional Animal Care and Use Committee of Yunnan Agricultural University with respect to ethical issues and scientific care.

### Oral glucose tolerance test.

The oral glucose tolerance test (OGTT) was performed in week 9. The mice were fasted overnight for 12 h and then given a glucose load (2 g/kg·BW). Blood glucose was measured before (0 min) and after (15, 30, 60, 90, and 120 min) glucose administration.

### Blood and tissue sample collection.

After the mice were sacrificed, the thoracic cavity was opened, and whole blood was taken from the abdominal aorta. The blood samples were centrifuged at 4,000 × *g* for 10 min at 4°C to obtain serum. Subcutaneous and visceral fat pads, liver, clean intestines (jejunum, distal ileum, cecum, and proximal colon), and cecum contents were collected from each mouse, flash-frozen in liquid nitrogen within 10 min postmortem, and then stored in a −80°C freezer (70).

### Histopathological examination.

The liver, epididymal fat, distal ileum, and proximal colon were immediately removed and fixed in 10% neutral formaldehyde fixative at 4°C. Tissues were cut into 5-mm-thick sections embedded in paraffin. Paraffin sections of 3 μm were stained with hematoxylin and eosin (H&E). Images were captured with an Olympus CX43 microscope and CellSens Entry software. The ratio of the ballooning degeneration area to the percent coverage of the nucleus was determined using an image analyzer (Image pro-Plus 6.0; Media Cybernetics, Inc., USA). Measurements were made on liver, epididymal fat, colon, or ileum sections from at least 10 independent pictures per mouse.

### RNA preparation and quantitative PCR analysis of gene expression.

Total RNA extraction and quantitative reverse transcription PCR (RT-qPCR) analysis of gene expression were performed using a method described previously (70). The primer sequences are presented in Table S1 in the supplemental material.

### Western blot analysis.

Thirty milligrams of distal ileum or proximal colon was lysed in 300 μl of radioimmunoprecipitation assay (RIPA) buffer (Strong, catalog number E121-01; Genstar, China) containing phenylmethylsulfonyl fluoride (PMSF) (1 mM) and then homogenized. The protein concentration was determined using a bicinchoninic acid (BCA) protein kit (Beyotime Biotechnology, China). Whole amounts of protein (60 μg) were loaded onto a 10% or 5% acrylamide gel, resolved in SDS-PAGE systems, and then transferred onto polyvinylidene difluoride (PVDF) membranes (0.45 μm; Millipore). The membranes were blocked in 5% skimmed milk powder, incubated with the primary antibodies overnight at 4°C, and then incubated with the secondary antibody for 1 h at 37°C. After the membranes were washed 3 times, the protein bands were visualized using an enhanced chemiluminescence kit (Tiangen Biotech, Beijing, China) according to the manufacturer’s instructions. Protein levels were normalized to β-actin as a loading control.

### Sequencing of 16S rRNA genes of gut microbiota and bioinformatics analysis.

Metagenomic DNA was extracted from the cecal contents using a QIAamp-DNA stool minikit (Qiagen, Hilden, Germany) according to the manufacturer’s instructions. For 16S rRNA gene sequencing, the DNA samples were sent to Majorbio Biotechnology Co., Ltd. (Shanghai, China), under dry ice conditions. The DNA concentration and purity were determined using a NanoDrop 2000 UV-visible (UV-Vis) spectrophotometer (Thermo Scientific, Wilmington, DE, USA), and DNA quality was checked by 1% agarose gel electrophoresis. The V3-V4 hypervariable regions of the bacterial 16S rRNA gene were amplified with primers 338F (5′-ACTCCTACGGGAGGCAGCAG-3′) and 806R (5′-GGACTACHVGGGTWTCTAAT-3′) using a thermocycler PCR system (GeneAmp 9700; ABI, USA). The PCRs were conducted using the following program: 3 min of denaturation at 95°C; 27 cycles of 30 s at 95°C, 30 s for annealing at 55°C, and 45 s for elongation at 72°C; and a final extension step at 72°C for 10 min. PCRs were performed in triplicate in a 20-μl mixture containing 4 μl of 5× FastPfu buffer, 2 μl of 2.5 mM deoxynucleoside triphosphates (dNTPs), 0.8 μl of each primer (5 μM), 0.4 μl of FastPfu polymerase, and 10 ng of template DNA. The resulting PCR products were extracted from a 2% agarose gel, purified using the AxyPrep DNA gel extraction kit (Axygen Biosciences, Union City, CA, USA), and quantified using QuantiFluor-ST (Promega, USA) according to the manufacturer’s protocol. Purified amplicons were pooled in equimolar amounts and paired-end sequenced (2 by 300) on an Illumina MiSeq PE300 platform (Illumina, San Diego, CA, USA) according to standard protocols by Majorbio Bio-Pharm Technology Co., Ltd. (Shanghai, China). All of the results were based on sequenced reads and operational taxonomic units (OTUs). The taxonomy of each 16S rRNA gene sequence was analyzed by the RDP Classifier algorithm against the Silva (SSU132) 16S rRNA database using a confidence threshold of 70%. Subsequent bioinformatics analysis was performed through the cloud platform of Majorbio Bio-Pharm Technology Co., Ltd. The details of these methods are described in Text S1 in the supplemental material.

10.1128/mSystems.00391-20.1 TEXT S1 Supplemental materials and methods. Download Text S1, DOCX file, 0.02 MB. Copyright © 2020 Gao et al. 2020 Gao et al. This content is distributed under the terms of the Creative Commons Attribution 4.0 International license.

### Statistical analysis.

The data are expressed as means ± standard errors of the means (SEM). One-way analysis of variance (ANOVA) was performed to identify significant differences among three or more groups followed by the indicated *post hoc* test (Student-Newman-Keuls comparison test). The unpaired two-tailed Student *t* test was performed to analyze two independent groups. Bivariate correlations were calculated using Spearman’s *r* coefficients. Multivariate analyses, i.e., 3D-PCoA (three-dimensional principal-coordinate analysis), linear discriminant analysis effect size (LEfSe) analyses, and network analysis, were performed using the cloud platform of Majorbio Bio-Pharm Technology Co., Ltd. Heat maps were constructed using HemI 1.0 software (http://hemi.biocuckoo.org/down.php). Unless otherwise specified in the figure legends, the results were considered statistically significant at a *P* value of *≤*0.05.

### Data availability.

The raw reads of 16S rRNA gene sequence data were deposited in the NCBI Sequence Read Archive (SRA) database under BioProject accession number PRJNA628813.
