# Supplementary figures and images for: Correlations between α-Linolenic Acid-Improved Multitissue Homeostasis and Gut Microbiota in Mice Fed a High-Fat Diet
Source: mSystems. 2020 Nov 3;5(6):e00391-20. doi: 10.1128/mSystems.00391-20 (PMC7646523; doi:10.1128/mSystems.00391-20)

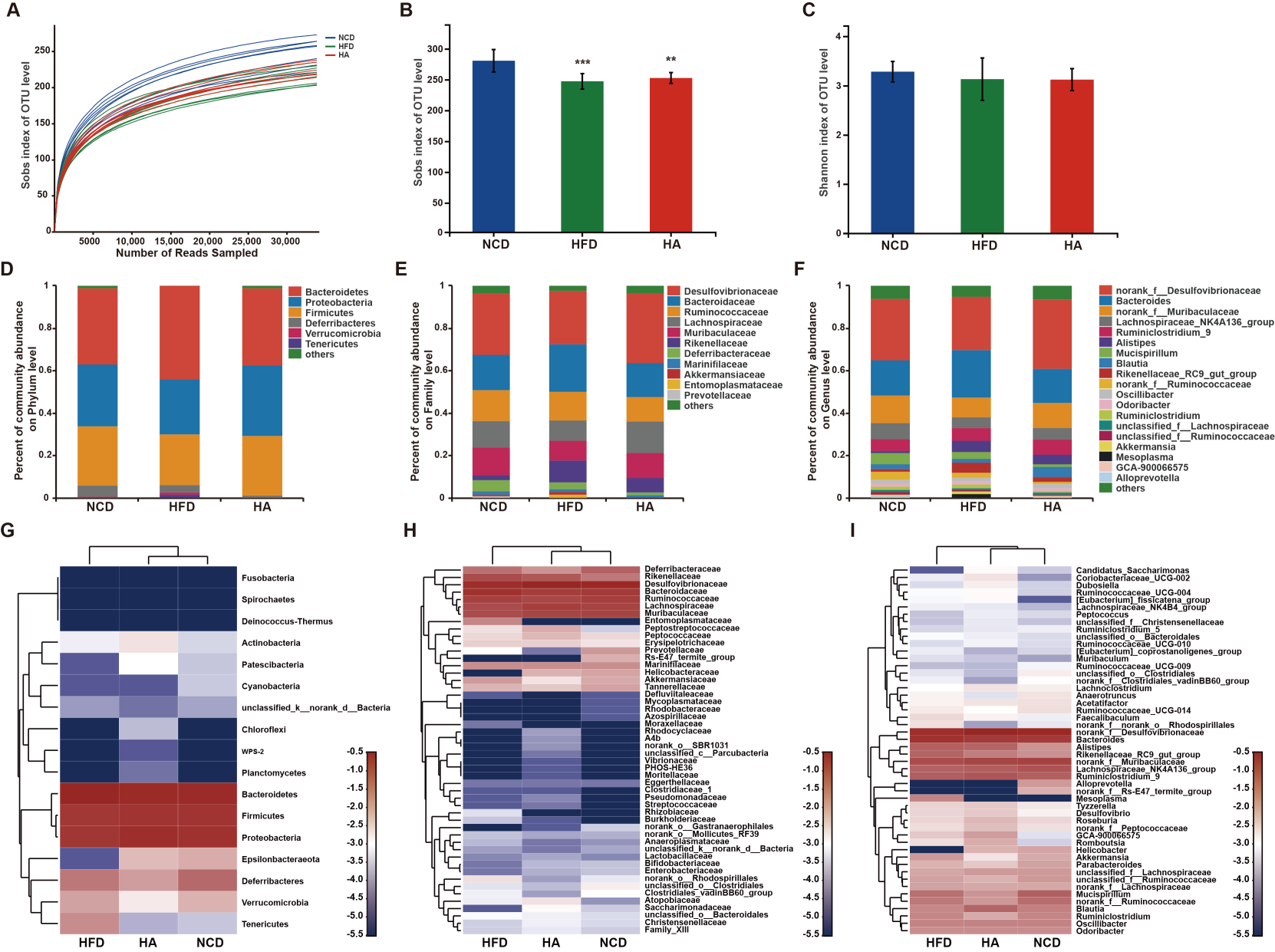

Supplement: FIG S1 [file mSystems.00391-20-sf001.docx]

**
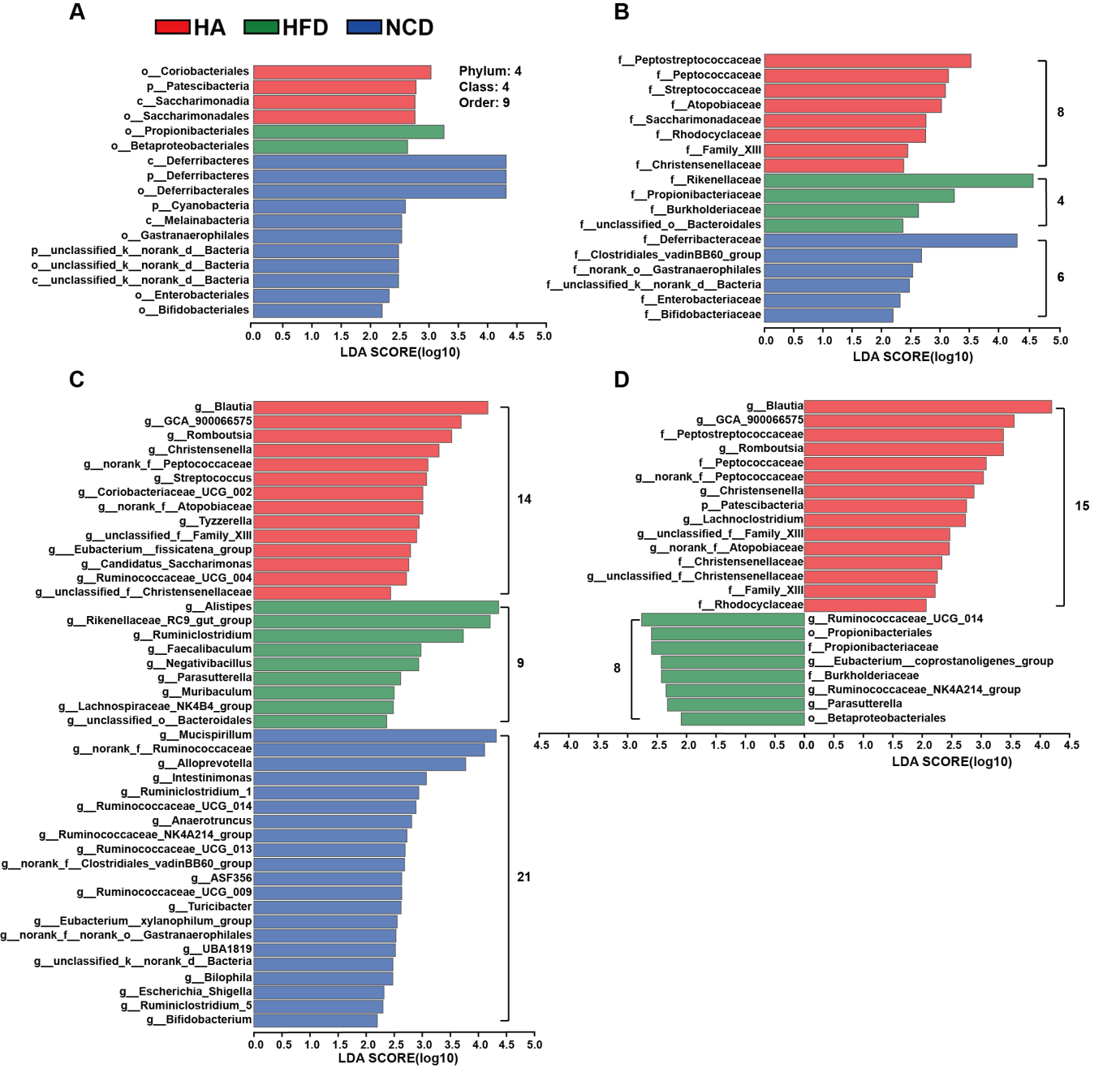
**

Supplement: FIG S2 [file mSystems.00391-20-sf002.docx]

**
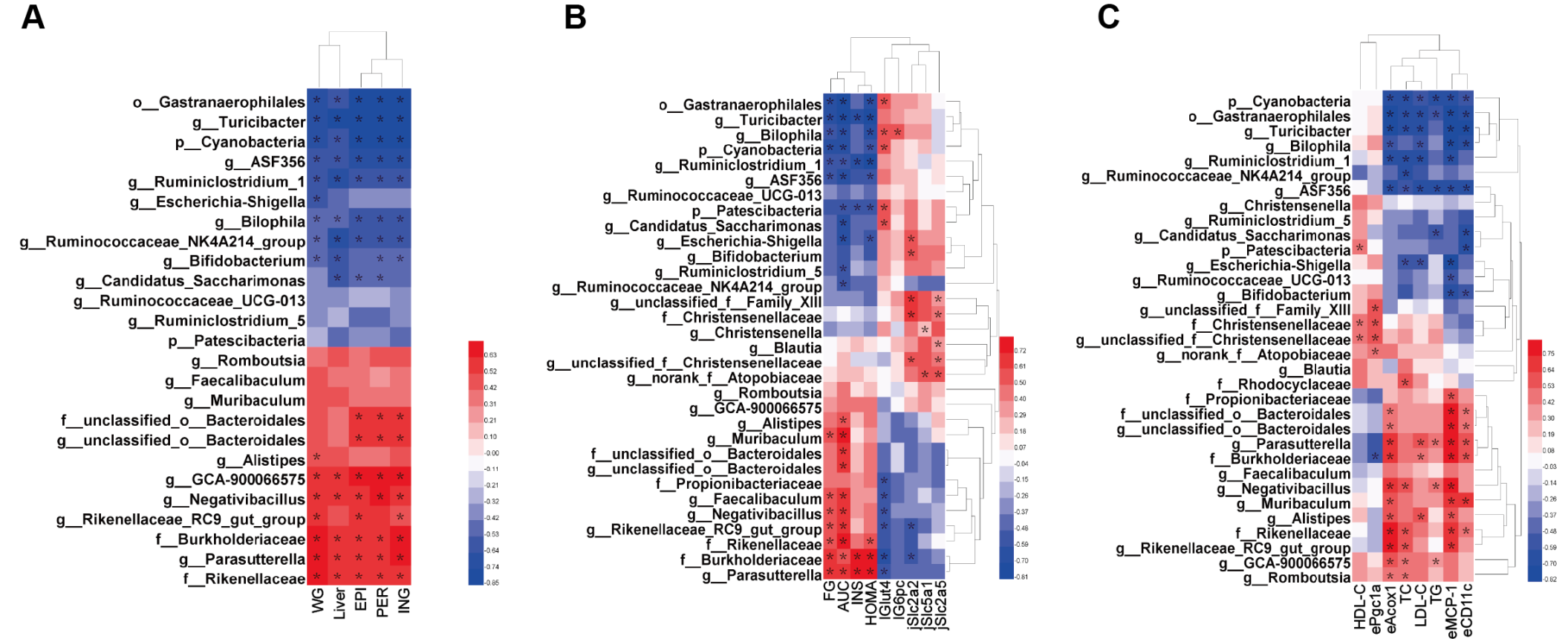
**

Supplement: FIG S3 [file mSystems.00391-20-sf003.docx]
