# Supplementary material for: Correlations between α-Linolenic Acid-Improved Multitissue Homeostasis and Gut Microbiota in Mice Fed a High-Fat Diet
Source: mSystems. 2020 Nov 3;5(6):e00391-20. doi: 10.1128/mSystems.00391-20 (PMC7646523; doi:10.1128/mSystems.00391-20)
Supplement: TABLE S1 [file mSystems.00391-20-st001.docx]

| Primer | Forward sequence | Reverse sequence |
| --- | --- | --- |
| *RPL-19* | GAAGGTCAAAGGGAATGTGTTCA | CCTTGTCTGCCTTCAGCTTGT |
| *Glut4* | ACGACGGACACTCCATCTGTTG | GGAGACATAGCTCATGGCTGGAA |
| *G6pc* | AGGAAGGATGGAGGAAGGAA | TGGAACCAGATGGGAAAGAG |
| *Slc2a1* | GGATCCCAGCAGCAAGAAG | CCAGTGTTATAGCCGAACTGC |
| *Slc2a2* | GTCAGCTATTCATCCACATTCAGT | AGCCAAGGTTCCGGTGAT |
| *Slc2a5* | AGAGCAACGATGGAGGAAAA | CCAGAGCAAGGACCAATGTC |
| *Slc5a1* | CTGGCAGGCCGAAGTATG | TTCCAATGTTACTGGCAAAGAG |
| *Sgk1* | GGACTACATTAATGGTGGAGAGC | CTGGCTATTTCAGCTGCGTA |
| *Acc1* | TGTTGAGACGCTGGTTTGTAGAA | GGTCCTTATTATTGTCCCAGACGTA |
| *Acox1* | CTATGGGATCAGCCAGAAAGG | AGTCAAAGGCATCCACCAAAG |
| *Pgc1α* | AGCCGTGACCACTGACAACGAG | GCTGCATGGTTCTGAGTGCTAAG |
| *Pparα* | CAACGGCGTCGAAGACAAA | TGACGGTCTCCACGGACAT |
| *Pparγ* | TCGCTGATGCACTGCCTATG | GAGAGGTCCACAGAGCTGATT |
| *TNF-α* | AGACCCTCACACTCAGATCA | TCTTTGAGATCCATGCCGTTG |
| *IL-1β* | TCCATGAGCTTTGTACAAGGA | AGCCCATACTTTAGGAAGACA |
| *IL-6* | GTTCTCTGGGAAATCGTGGA | TGTACTCCAGGTAGCTA |
| *MCP-1* | TTAAAAACCTGGATCGGAACCAA | GCATTAGCTTCAGATTTACGGGT |
| *IL-10* | AAGGACCAGCTGGACAACAT | TCTCACCCAGGGAATTCAAA |
| *INF-γ* | ATCTGGAGGAACTGGCAAAA | TTCAAGACTTCAAAGAGTCTGAGGTA |
| *CD11* | ACGTCAGTACAAGGAGATGTTGGA | ATCCTATTGCAGAATGCTTCTTTACC |
| *Reg3g* | TTCCTGTCCTCCATGATCAAA | CATCCACCTCTGTTGGGTTC |
| *Lyz1* | GCCAAGGTCTACAATCGTTGTGAGTTG | CAGTCAGCCAGCTTGACACCACG |
| *Pla2g2* | AGGATTCCCCCAAGGATGCCAC | CAGCCGTTTCTGACAGGAGTTCTGG |
| *Defa* | GGTGATCATCAGACCCCAGCATCAGT | AAGAGACTAAAACTGAGGAGCAGC |
| *Occludin* | ATGTCCGGCCGATGCTCTC | TTTGGCTGCTCTTGGGTCTGTAT |
| *ZO-1* | TTTTTGACAGGGGGAGTGG | TGCTGCAGAGGTCAAAGTTCAAG |
| *Muc2* | ACGTGTCATATTTGCACCTCT | TCAACATTGAGAGTGCCAACT |
| *TLR4* | GCAGAAAATGCCAGGATGATG | AACTACCTCTATGCAGGGATTCAAG |
| *CD14* | TCAGCTAAACTCGCTCAATC | TCCAGCCTGTTGTAACTGAG |
| *CB1* | CTGATGTTCTGGATCGGAGTC | TCTGAGGTGTGAATGATGATGC |
