# Supplementary material for: Correlations between α-Linolenic Acid-Improved Multitissue Homeostasis and Gut Microbiota in Mice Fed a High-Fat Diet
Source: mSystems. 2020 Nov 3;5(6):e00391-20. doi: 10.1128/mSystems.00391-20 (PMC7646523; doi:10.1128/mSystems.00391-20)
Supplement: TEXT S1 [file mSystems.00391-20-s0001.docx]

**Supplementary materials and methods**

**Blood and tissue sample collection.** After the mice were sacrificed, the thoracic cavity was opened, and whole blood was taken from the abdominal aorta. The blood samples were centrifuged at 4000 *g* for 10 min at 4°C to obtain the serum. The cecum contents were collected at the day of mouse dissection. Specifically, cecum contents were washed from cecum in a 2-mL Eppendorf tube containing 1.0 mL cold sterile UP water. All contents in the cecum of each mouse were collected. Subcutaneous and visceral fat pads, liver, clean intestines (jejunum, distal ileum, cecum, and proximal colon) and cecum contents were collected from each mouse, flash-frozen in liquid nitrogen within 10 min postmortem, and then stored in a -80°C freezer.

**DNA extraction and PCR amplification.**  Metagenomic DNA was extracted from the cecal content using a QIAamp-DNA stool mini-kit (Qiagen, Hilden, Germany) according to the manufacturer’s instructions. For 16S rRNA gene sequencing, the DNA samples were sent to Majorbio Biotechnology Co., Ltd. (Shanghai, China) under dry ice conditions. The DNA concentration and purification were determined by a NanoDrop 2000 UV-Vis spectrophotometer (Thermo Scientific, Wilmington, USA), and DNA quality was checked by 1% agarose gel electrophoresis. The hypervariable region V3-V4 of the bacterial 16S rRNA gene were amplified with primer pairs 338F (5'-ACTCCTACGGGAGGCAGCAG-3') and 806R (5'-GGACTACHVGGGTWTCTAAT-3') by an ABI GeneAmp® 9700 PCR thermocycler (ABI, CA, USA). The PCR amplification of 16S rRNA gene was performed as follows: initial denaturation at 95 ℃ for 3 min, followed by 27 cycles of denaturing at 95 ℃ for 30 s, annealing at 55 ℃ for 30 s and extension at 72 ℃ for 45 s, and single extension at 72 ℃ for 10 min, and end at 4 ℃. The PCR mixtures contain 5 × TransStart FastPfu buffer 4 μL, 2.5 mM dNTPs 2 μL , forward primer (5 μM) 0.8 μL, reverse primer (5 μM) 0.8 μL, TransStart FastPfu DNA Polymerase 0.4 μL, template DNA 10 ng, and finally ddH2O up to 20 μL. PCR reactions were performed in triplicate. The resulting PCR products were extracted from a 2% agarose gel, purified using the AxyPrep DNA Gel Extraction Kit (Axygen Biosciences, Union City, CA, USA) and quantified using QuantiFluor™-ST (Promega, USA) according to the manufacturer’s protocol.

**Illumina MiSeq sequencing.** Purified amplicons were pooled in equimolar and paired-end sequenced on an Illumina MiSeq PE300 platform (Illumina, San Diego,USA) according to the standard protocols by Majorbio Bio-Pharm Technology Co. Ltd. (Shanghai, China). The raw reads were deposited into the NCBI Sequence Read Archive (SRA) database (Accession Number: PRJNA628813.).

**Processing of sequencing data.** The raw 16S rRNA gene sequencing reads were demultiplexed, quality-filtered by fastp version 0.20.0^[1]^ and merged by FLASH version 1.2.7^[2]^ with the following criteria: (i) the 300 bp reads were truncated at any site receiving an average quality score of < 20 over a 50 bp sliding window, and the truncated reads shorter than 50 bp were discarded, reads containing ambiguous characters were also discarded; (ii) only overlapping sequences longer than 10 bp were assembled according to their overlapped sequence. The maximum mismatch ratio of overlap region is 0.2. Reads that could not be assembled were discarded; (iii) Samples were distinguished according to the barcode and primers, and the sequence direction was adjusted, exact barcode matching, 2 nucleotide mismatch in primer matching.

Operational taxonomic units (OTUs) with 97% similarity cutoff ^[3, 4]^ were clustered using UPARSE version 7.1^[3]^, and chimeric sequences were identified and removed. The taxonomy of each OTU representative sequence was analyzed by RDP Classifier version 2.2^[5]^ against the 16S rRNA database using confidence threshold of 0.7.

Purified amplicons were pooled in equimolar amounts and paired-end sequenced (2 × 300) on an Illumina MiSeq platform (Illumina, San Diego, CA, USA) according to the standard protocols by Majorbio Bio-Pharm Technology Co. Ltd. (Shanghai, China). All of the results were based on sequenced reads and operational taxonomic units (OTUs). The taxonomy of each 16S rRNA gene sequence was analyzed by the RDP Classifier algorithm against the Silva (SSU132) 16S rRNA database using a confidence threshold of 70%. Subsequent bioinformatics analysis was performed through the cloud platform of Majorbio Bio-Pharm Technology Co. Ltd.

**References:**

[1] Chen S, Zhou Y, Chen Y, Gu J. fastp: an ultra-fast all-in-one FASTQ preprocessor. Bioinformatics. 2018, 34(17): i884‐i890. doi:10.1093/bioinformatics/bty560.

[2] Magoč T, Salzberg SL. FLASH: fast length adjustment of short reads to improve genome assemblies. Bioinformatics. 2011, 27(21):2957‐2963. doi:10.1093/bioinformatics/btr507

[3] Edgar RC. UPARSE: highly accurate OTU sequences from microbial amplicon reads. Nat Methods. 2013, 10(10):996‐998. doi:10.1038/nmeth.2604.

[4] Stackebrandt E , Goebel B M . Taxonomic Note: A Place for DNA-DNA Reassociation and 16S rRNA Sequence Analysis in the Present Species Definition in Bacteriology[J]. International Journal of Systematic Bacteriology, 1994, 44(4):846-849. doi:10.1099/00207713-44-4-846.

[5] Wang Q, Garrity GM, Tiedje JM, Cole JR. Naive Bayesian classifier for rapid assignment of rRNA sequences into the new bacterial taxonomy. Appl Environ Microbiol. 2007, 73(16):5261‐5267. doi:10.1128/AEM.00062-07.
